# PDGF Receptor Alpha Signaling Is Key for Müller Cell Homeostasis Functions

**DOI:** 10.3390/ijms22031174

**Published:** 2021-01-25

**Authors:** Nundehui Díaz-Lezama, Anne Wolf, Susanne Koch, Anna M. Pfaller, Josef Biber, Xavier Guillonneau, Thomas Langmann, Antje Grosche

**Affiliations:** 1 Department of Physiological Genomics, Biomedical Center, Ludwig-Maximilians-Universität München, D-82152 Planegg-Martinsried, Germany; nundehui.diaz_lezama@tu-dresden.de (N.D.-L.); Susanne.Koch@bmc.med.lmu.de (S.K.); Anna.Pfaller@bmc.med.lmu.de (A.M.P.); Josef.Biber@bmc.med.lmu.de (J.B.); 2 Laboratory for Experimental Immunology of the Eye, Department of Ophthalmology, Faculty of Medicine and University Hospital Cologne, University of Cologne, D-50931 Cologne, Germany; anne.wolf@uk-koeln.de (A.W.); thomas.langmann@uk-koeln.de (T.L.); 3 Institut de la Vision, INSERM, CNRS, Sorbonne Université, F-75012 Paris, France; xavier.guillonneau@inserm.fr

**Keywords:** Müller cells, retina, PDGF receptor alpha, choroidal neovascularization, gliosis

## Abstract

Müller cells, the major retinal macroglia, are key to maintaining vascular integrity as well as retinal fluid and ion homeostasis. Although platelet derived growth factor (PDGF) receptor expression in Müller glia has been reported earlier, their actual role for Müller cell function and intimate interaction with cells of the retinal neurovascular unit remains unclear. To close this gap of knowledge, Müller cell-specific PDGF receptor alpha (PDGFRα) knockout (KO) mice were generated, characterized, and subjected to a model of choroidal neovascularization (CNV). PDGFRα-deficient Müller cells could not counterbalance hypoosmotic stress as efficiently as their wildtype counterparts. In wildtypes, the PDGFRα ligand PDGF-BB prevented Müller cell swelling induced by the administration of barium ions. This effect could be blocked by the PDGFR family inhibitor AC710. PDGF-BB could not restore the capability of an efficient volume regulation in PDGFRα KO Müller cells. Additionally, PDGFRα KO mice displayed reduced rod and cone-driven light responses. Altogether, these findings suggest that Müller glial PDGFRα is central for retinal functions under physiological conditions. In contrast, Müller cell-specific PDGFRα KO resulted in less vascular leakage and smaller lesion areas in the CNV model. Of note, the effect size was comparable to pharmacological blockade of PDGF signaling alone or in combination with anti-vascular endothelial growth factor (VEGF) therapy—a treatment regimen currently being tested in clinical trials. These data imply that targeting PDGF to treat retinal neovascular diseases may have short-term beneficial effects, but may elicit unwarranted side effects given the putative negative effects on Müller cell homeostatic functions potentially interfering with a long-term positive outcome.

## 1. Introduction

The effect of platelet-derived growth factor (PDGF) upon the stabilization of blood vessel integrity mediated via the activation of its specific receptors, PDGF receptor α (PDGFRα) and β (PDGFRβ), was intensively investigated throughout the past years. These studies point to a central role of PDGF in recruiting pericytes to newly built blood vessels in the course of physiological angiogenesis during retinal development, but also in the context of pathological neovascularization as it occurs in diabetic retinopathy and age-related macular degeneration [1,2,3,4].The gold standard therapy of these diseases with medium-term success currently is the intravitreal injection of antibody derivates directed against vascular endothelial growth factor (VEGF; [5]). It has been proposed that treatment strategies combining anti-VEGF therapy with agents blocking PDGF signaling should be even more effective than the respective single treatment regimen [6,7]. The hypothesis behind it is that hindering PDGF signaling prevents pericyte–endothelial interactions in the process of neovessel formation, thereby reducing growth of the latter as well as associated problems such as scar formation, vascular leakage, and edema formation [8]. The aforementioned combinatory therapy has already been under investigation for its beneficial effects in clinical testing (e.g., phase III trials—https://clinicaltrials.gov/ identifiers: NCT01944839, NCT01940900, NCT01940887; [9]), but there remain several open questions regarding the effects of the anti-PDGF therapy at the cellular level. For example, it should be analyzed whether PDGF—similar to what was shown for VEGF [10]—is essential to the survival of different cell types of the retina. This kind of investigation allows for conclusions about putative (probably unwarranted) side effects of a long-term anti-PDGF therapy.

Immunofluorescence studies on the expression of PDGF-BB, PDGFRα, and PDGFRβ suggest high expression levels in vascular and Müller cells [11,12,13]. Given that Müller cells are intimately interacting with pericytes and endothelial cells via perivascular processes [14,15], an intense PDGF-mediated crosstalk of these cellular entities in the course of angiogenesis and maintenance of vessel integrity is very likely. Supporting this assumption, Ikuno et al. [16] demonstrated that Müller cells could stimulate the proliferation of pericytes in vitro by secreting PDGF. That Müller cells are indeed essential to maintaining intact blood vessels was shown by Shen et al. [17] with an elegant transgenic mouse model that allows selective, time-controlled ablation of Müller cells in vivo. Loss of the latter leads to retinal disorganization, including vascular anomalies. Interestingly, in a human pathology—namely, idiopathic macular telangiectasia type 2—Müller cells appear to be absent from the central retina and there are speculations that the retinal edema associated with dilated, leaky vessels frequently found in the macula of these patients is due to the aforementioned Müller cell loss [18,19]. 

Several mouse models have been developed to investigate the functional role of PDGF signaling. Expression of binding-deficient PDGF-B instead of its intact counterpart resulted in malformed vessels with detached pericytes not getting into contact with the endothelial cells [20]. If PDGF was overexpressed, pericytes and endothelial and Müller cells were strongly proliferating, thereby promoting retinal degeneration [21,22,23]. However, even though expression of PDGF receptors on Müller cells was demonstrated years ago, almost nothing is known about the actual role of PDGF for Müller cell function and its interaction via this signaling pathway with other retinal cell types. Here we collected pioneer data about the role of PDGF signaling for Müller cells in vivo by use of a time-controlled Müller cell-specific PDGFRα knockout in mice combined with a model of choroidal neovascularization, addressing the question of whether Müller cells contribute to the modulation of vascular integrity via PDGF signaling. 

## 2. Results

### 2.1. Induced Müller Cell-Specific Knockout of PDGFRα 

**Figure 1:**
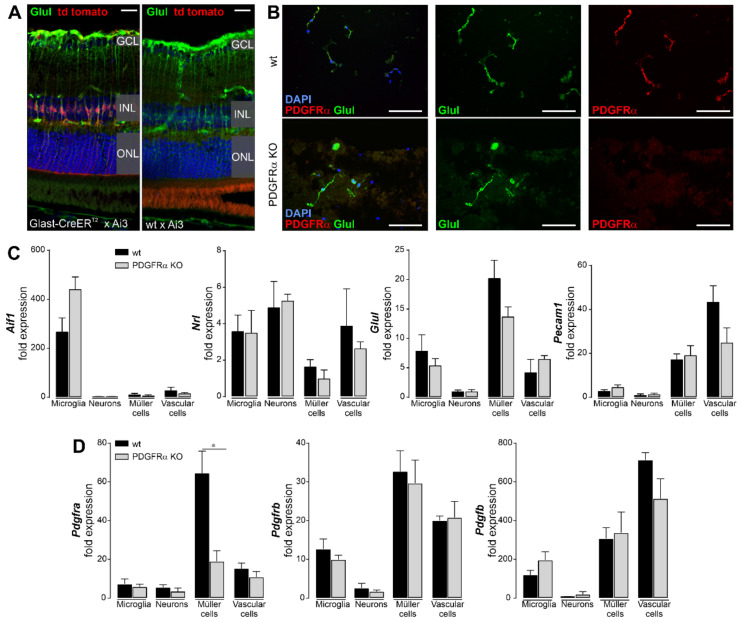
Validation of Müller cell-specific deletion of PDGFRα in tamoxifen-injected B6.Cg-Pdgfra^tm8Sor^/EiJ; B6.Cg-Glast-CreER^T2^ double transgenic mouse line 4 weeks post-injection. (**A**) Retinal cryosections from B6.Cg-Glast-CreER^T2^ mice that were crossbred with Ai3 mice [24]. The mice express tdTomato as the reporter for Cre recombinase activity. Note that tamoxifen injection led to expression of tdTomato in virtually every Müller cell (co-stained for the marker glutamine synthetase, Glul) and not in any other retinal cell type, demonstrating that the Glast-CreER^T2^ very efficiently and specifically drives expression of Cre recombinase in Müller glia. Scale bar, 20 µm. GCL, ganglion cell layer; INL, inner nuclear layer; ONL, outer nuclear layer. (**B**) Representative micrographs from Müller cells purified from retinae of respective genotypes and labeled for PDGFRα (*red*) and co-stained for the Müller cell marker glutamine synthetase (Glul, *green*). Note the absence of PDGFRα from Müller cells of B6.Cg-Pdgfra^tm8Sor^/EiJ; B6.Cg-Glast-CreER^T2^ (PDGFRα KO mice) double transgenic mice. Nuclei were visualized by DAPI co-staining. Scale bars, 100 µm. (**C**) Validation of enrichment of various retinal cell populations for cell type-specific expression analysis via magnetic activated cell sorting. Marker genes for microglia (*aif1*), rods as the most abundant neuron of a mouse retina (*nrl*), Müller cells (*glul*), and vascular cells (*pecam1*) were analyzed by quantitative real-time PCR (qPCR). (**D**) Knockout of PDGFRα was validated via qPCR on retinal cell populations purified by magnetic activated cell sorting. *Pdgfra* was specifically expressed by Müller cells and its expression was downregulated to ~30% in Müller cells of PDGFRα KO mice. No effect on *pdgfra* expression levels was observed in the other retinal cell populations. Similarly, *pdgfrb* and *pdgfb* expression levels were not affected by Cre recombinase expression in Müller cells of PDGFRα KO mice. Bars represent mean ± SEM and data were collected from 3–5 animals. * *p* < 0.05.

From initial experiments, we determined that the Glast-CreER^T2^ construct drives very efficient Cre recombinase expression in mice crossbred with the Ai3 reporter line (Figure 1A, [24]). After recombination induced by tamoxifen administration, virtually every Müller cell expressed the reporter tdTomato. Importantly, reporter expression in other retinal cell types was not observed. This proved the usability of the Glast-CreER^T2^ construct to mediate Müller cell-specific knockout via the Cre recombinase system. To validate the efficiency of PDGFRα ablation in Müller glia, PDGFRα expression was detected via immunostaining. The vast majority of isolated Müller cells from PDGFRα KO mice was lacking immunoreactivity for PDGFRα. In contrast, the Müller cell fraction obtained from wildtype littermates showed a strong expression of the receptor (Figure 1B). PDGFRα staining of Müller cell inner stem processes and vascular structures was observed in retinal cross-sections, which was largely absent from the PDGFRα KO retina (Figure S1). Next, we isolated RNA from retinal cell populations purified by magnetic activated cell sorting as described recently [25,26] and performed quantitative real-time PCR (qPCR) to further evaluate the PDGFRα KO efficiency and specificity. Enrichment of the various retinal cell populations was validated on the basis of high expression levels of respective marker genes (Figure 1C). Even though there were some obvious cross contaminations, such as photoreceptors (marker gene neural retina leucine zipper, *nrl*) being present in the microglia and vascular cell populations, Müller cells (expressing the marker gene glutamine synthetase, *glul*), on which we focus here, were clearly purified and rather free from microglia (marker gene *aif1*) and photoreceptors (*nrl*) and at least partially separated from vascular cells expressing *pecam1*. In the end, the qPCR data confirmed a highly specific *pdgfra* expression in Müller cells compared to other retinal cell populations (Figure 1D). In line with the findings from the histological analysis, mRNA levels of Pdgfra were significantly lower in the Müller cell population from PDGFRα KO mice (about 30% of the expression found in wildtypes). Importantly, the *pdgfra* transcript level in other retinal cell types was not affected by tamoxifen-induced Müller cell-specific PDGFRα knockout (Figure 1D). In sum, these results corroborated the cell type specificity and efficiency of the PDGFRα KO via the Glast-CreER^T2^ driver line. 

Finally, we tested for potential expression changes of other components of the PDGF pathway upon PDGFRα knockout in Müller cells. Transcript levels of *pdgfrb* and of the ligand *pdgfb* showed no compensatory up- or downregulation in any retinal cell type (Figure 1D). *Pdgfrb* was expressed in a similar amount in microglia, Müller cells, and vascular cells, whereas Pdgfb was mainly expressed by vascular cells and at lower levels in Müller cells and microglia. Of note, PDGF signaling did not seem to directly involve retinal neurons, as they presented with very low expression levels of receptors and the ligand (Figure 1D).

### 2.2. Normal Retinal Morphology in Müller Cell-Specific PDGFRα KO Mice

**Figure 2:**
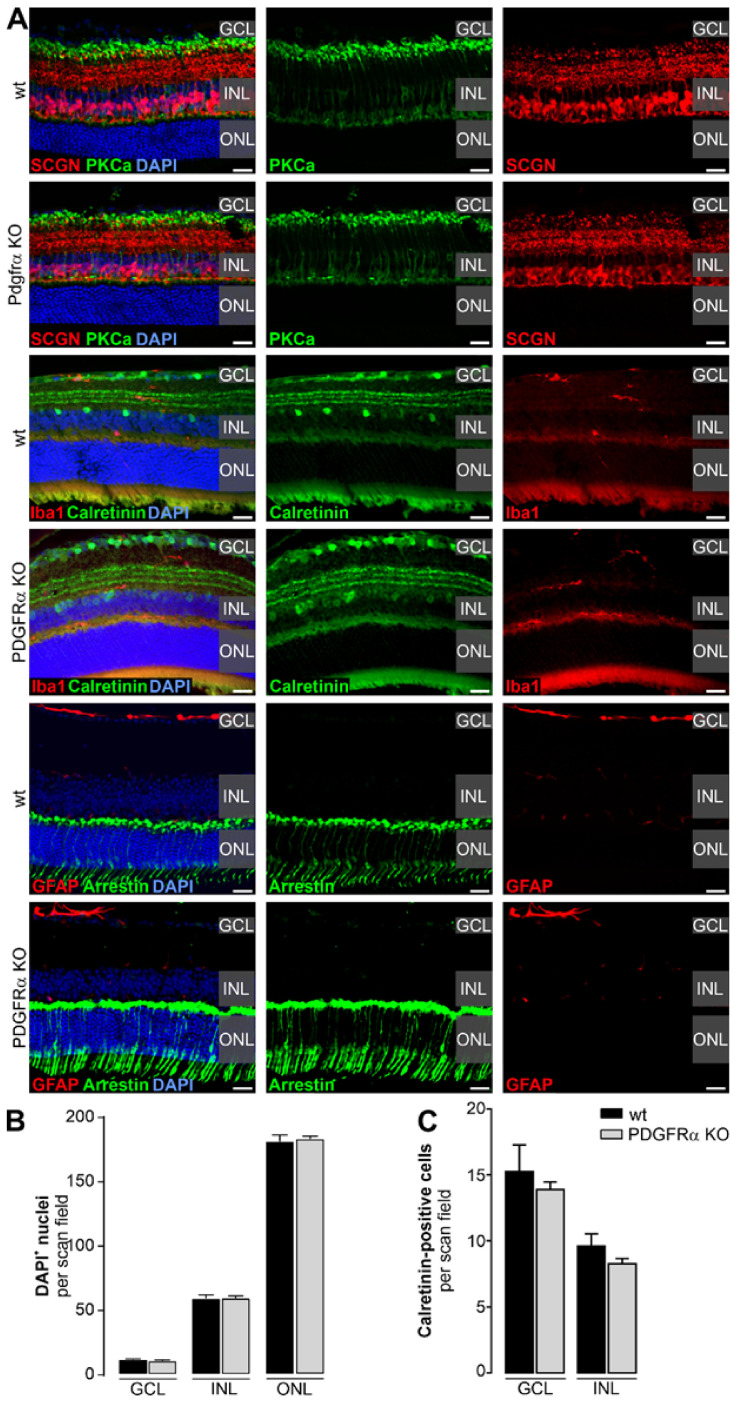
Characterization of retinal architecture in Müller cell-specific PDGFRα KO mice. (**A**) Various retinal cell types in Müller cell-specific PDGFRα KO mice were characterized regarding cell counts and morphology to those of wildtype (wt) retinae using cell type-specific markers. Representative scans are shown. Specifically, cone bipolar cells (secretagogin, SCGN), rod bipolar cells (PKCα), microglia (Iba1), ganglion and amacrine cells (calretinin), astrocytes and reactive Müller cells (glial fibrillary acidic protein, GFAP), and cones (arrestin) were examined. Nuclei were visualized by DAPI co-staining. Scale bars, 20 µm. ONL, outer nuclear layer. (**B**) Morphometric analysis was performed on central retinal slices of wt and PDGFRα KO mice. Cell numbers from the three nuclear layers were assessed by quantification of DAPI^+^ nuclei. No difference could be observed between wt and PDGFRα KO retinae. Bars represent mean ± SEM and data were collected from four animals of each genotype. (**C**) Central retinal sections stained for calretinin, a marker of amacrine cells in the inner nuclear layer (INL) and of ganglion and displaced amacrine cells in the ganglion cell layer (GCL), were used to quantify cell numbers between the different genotypes. Bars represent mean ± SEM and data were collected from 3–5 animals of each genotype.

To analyze whether the disruption of the PDGFRα-related signaling in Müller glia affects retinal histology, we carefully evaluated the retinal phenotype in the Müller cell-specific PDGFRα KO mice. To assess the preservation of the retinal microarchitecture, morphometric analysis was performed (Figure 2, Figure S2). No differences regarding cell numbers quantified on the basis of DAPI staining for any of the three nuclear layers were found compared to the wildtype retinae (Figure 2B). In line with this, TUNEL labeling revealed that neither in wildtype nor in PDGFRα KO retinae did cells undergo DNA fragmentation as a sign of apoptotic cell death (Figure S2B). Subsequently, we immunolabeled sections with different neuronal markers such as anti-secretagogin (SCGN) to evaluate cone bipolar cells and their dendrites and anti-protein kinase-C-α (PKCα) delineating rod bipolar and a subpopulation of amacrine cells (Figure 2A). SCGN- and PKCα-positive bipolar cells did not show any kind of morphological abnormalities in the PDGFRα KO mouse. In addition, we analyzed the number of calretinin-positive ganglion and amacrine cells in the GCL and INL, respectively. We did not find any changes in number between the retinae from the two genotypes, and the three layers of dendrites of calretinin-positive cells in the IPL appeared normal (Figure 2A,C). Next, we evaluated a possible effect of the PDGFRα knockout on photoreceptor integrity. Cone arrestin and PDE6b (rods) immunostaining did not reveal any major morphological alterations of the two photoreceptor types (Figure 2A and Figure S2A). The labeling pattern of glial fibrillary acidic protein (GFAP) was unchanged in PDGFRα KO mice. Accordingly, this marker delineated astrocytes in the nerve fiber layer, but no GFAP upregulation in Müller cells as a sign of gliotic changes was found (Figure 2A). Müller glia were additionally evaluated by immunostaining for glutamine synthetase to assess morphological alterations, but no differences were found when PDGFRα KO Müller cells were compared to their wildtype counterparts, either (Figure S2A). In addition, microglia, the resident tissue macrophages of the retina, did not display any morphological changes that would be indicative of their activation and, thus, of ongoing degenerative processes in the tissue (Figure 2A, Figure S2C). Finally, we also investigated the integrity of the retinal pigment epithelium (RPE) through β-catenin immunostaining, which delineates RPE membranes. No apparent changes in staining intensities or a disruption of the RPE monolayer were found in the PDGFRα KO mice (Figure S2D).

### 2.3. Functional Disruption in Retinae of Müller Cell-Specific PDGFRα KO Mice

**Figure 3:**
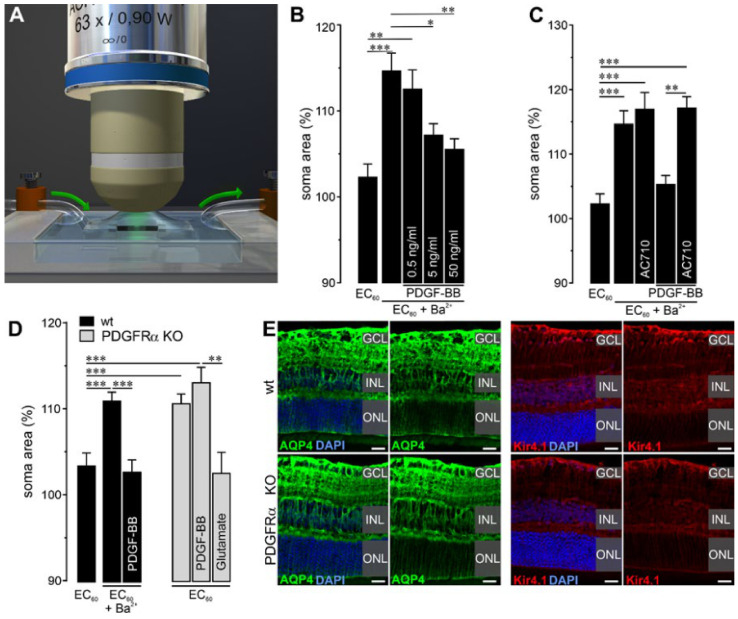
Dysfunctional volume homeostasis in PDGFRα-deficient Müller cells. (**A**) Scheme of the experimental setup to determine Müller cell volume regulation. Vital retinal slices were stained with Mitotracker Orange and were examined via confocal scanning microscopy while hypotonic solution was washed into the recording chamber. (**B**) In wildtype Müller cells, PDGF-BB showed a dose-dependent effect, preventing the swelling of Müller cells induced by the co-application of barium ions (1 mM, Ba^2+^) and exposure to 4 min of hypoosmotic stress (60% osmolarity). (**C**) AC710, a potent PDGFR family inhibitor (100 µM), abolished the protective action of PDGF-BB (50 ng/mL) in Müller cells from wildtype animals. (**D**) In the absence of PDGFRα, Müller cells were not able to counterbalance volume changes under hypoosmotic stress as efficiently as their wildtype counterparts. Administration of the PDGFRα ligand PDGF-BB (50 ng/mL) did not prevent cell volume changes in Müller cell-specific PDGFRα KO retinae. In contrast, glutamate (1 mM) efficiently blocked cell swelling of PDGFRα-deficient Müller cells under hypoosmotic stress. Experiments on wildtype littermates were repeated, combining barium ions and PDGF-BB to demonstrate no adverse effect of tamoxifen treatment on key findings observed in (**C**). (**B**–**D**) Bars represent mean ± SEM and data were collected from 15–34 cells isolated from 2–3 animals of each genotype. * *p* < 0.05, ** *p* < 0.01; *** *p* < 0.001. (**B**) * Changes in soma were tested after 4 min of exposure to hypoosmolar (60% osmolarity) extracellular solution (EC_60_) with a Mann–Whitney U-test. (**E**) Representative images of immunofluorescent detection of the water channel aquaporin 4 (AQP4) and the potassium channel Kir4.1 in Müller cell-specific PDGFRα KO retinae and wildtypes. Both proteins were enriched in Müller cell end feet facing the vitreous and blood vessels. There was no discernible phenotype in terms of immunoreactivity or distribution of Kir4.1 and AQP4. GCL, ganglion cell layer; INL, inner nuclear layer; ONL, outer nuclear layer. Scale bars, 20 µm.

The high expression level in Müller cells strengthened our assumption that PDGFRα might play a major role in their cellular functions. Accordingly, we addressed the effects of the knockout on Müller cell physiology directly. Recently, we demonstrated that growth factors such as VEGF acting via receptor tyrosine kinase receptors similar to PDGF are key for Müller cell volume regulation [27]. To test if PDGF acts in a comparable way on this Müller cell function, we measured their capability to counteract volume changes when subjected to hypoosmotic stress (Figure 3A). Typically, Müller cells do not swell under these conditions. However, if their potassium channels are blocked by the administration of barium ions, hypoosmotic stress immediately induces an increase in the Müller cell soma size, which is measured as an indicator for volume changes (Figure 3B). Co-application of PDGF-BB, a PDGFRα ligand, restored their efficient volume regulation in a dose-dependent manner (Figure 3B). This effect could be blocked by the rather unspecific PDGFR family inhibitor AC710 (Figure 3C). To circumvent drawbacks of unspecific pharmacological effects, we next checked whether knockout of PDGFRα would lead to disrupted Müller cell volume regulation. Indeed, PDGFRα-deficient Müller cells swelled immediately even in the absence of any other tissue damage or channel blockers such as barium ions (Figure 3D). Growth factors such as VEGF induce a complex glutamatergic–purinergic signaling cascade fostering efficient Müller cell volume regulation [27]. Accordingly, we checked whether glutamate, known to act downstream of those growth factors and, thus, possibly also downstream of PDGFRα, would reduce swelling in PDGFRα-deficient Müller cells. Indeed, no swelling of Müller cells in knockout retinae was observed upon glutamate administration under hypoosmotic conditions (Figure 3D).

In addition to the growth factor-induced glutamatergic–purinergic signaling cascade, the inwardly rectifying potassium channel Kir4.1, as well as the water channel protein aquaporin-4 (AQP4), were implicated in osmotic volume regulation in Müller cells [28,29,30]. If the absence of PDGFRα were to change their expression or spatial distribution, this could be an alternative explanation for our finding of disturbed Müller cell volume regulation. However, Müller cell-specific PDGFRα knockout retinae displayed a normal localization of Kir4.1 and AQP4 primarily in Müller cell end feet lining the inner retinal surface towards the vitreous, and in their processes, enwrapping blood vessels (Figure 3E). The intensity of the immunostaining was comparable between knockout and wildtype retinae. Altogether, these data suggest that (i) PDGF-mediated signaling is involved in Müller cell volume regulation and (ii) that PDGFRα deficiency cannot be compensated by PDGFRβ, at least not in the context of Müller cell volume regulation.

**Figure 4:**
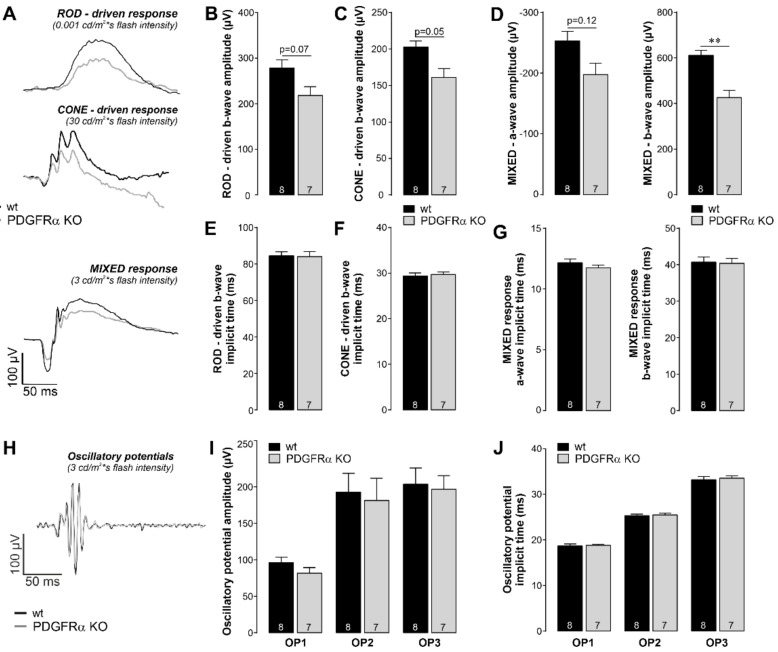
Altered retinal light responses from Müller cell-specific PDGFRα-KO mice. (**A**) Representative electroretinogram (ERG) waveforms for rods (upper), cones (middle), and mixed (lower) responses from wildtype (black) and PDGFRα KO (grey) mice. (**B**,**C**) ERG amplitudes for the scotopic rod-specific b-wave (**B**) and the photopic cone-specific b-wave (**C**). (**D**) ERG amplitudes for the mixed rod–cone-specific a-wave and the mixed rod–cone-specific b-wave. (**E**,**F**) Implicit times for the scotopic rod-specific b-wave (**E**) and the photopic cone-specific b-wave (**F**). (**G**) Implicit times for the mixed rod–cone-specific a-wave and the mixed rod–cone-specific b-wave. (**H**) Representative oscillatory potential waveforms for mixed responses. (**I**) Oscillatory potential amplitudes for the mixed rod–cone-specific response. (**J**) Oscillatory potential implicit times for the mixed rod–cone-specific response. Amplitudes and implicit times from the first, second, and third positive peaks of the oscillatory potentials were quantified and labeled as OP1, OP2, and OP3, respectively (**I**,**J**). Bars represent mean values ± SEM from 7 to 8 animals. ** *p* < 0.01, Mann–Whitney U-test. For each mouse, the two eyes were averaged.

Given our finding that Müller cell volume regulation was impaired in PDGFRα KO mice, we asked whether as a consequence retinal function would also be altered in those mice. Indeed, we found considerable functional deficits in the retinal light responses of Müller cell-specific PDGFRα KO mice as determined by electroretinogram (ERG) recordings (Figure 4A). Under dark-adapted conditions with dim flashes, to derive rod responses, a rod-specific b-wave (positive deflection) was recorded. The rod-driven b-wave amplitude was reduced in PDGFRα KO mice compared to wildtype, but the difference was not statistically significant (*p* = 0.07) (Figure 4B). Under light-adapted conditions with very bright flashes, to derive cone responses, a cone specific b-wave was recorded. Again, the cone-driven b-wave amplitude was decreased in PDGFRα KO mice (*p* = 0.05) (Figure 4C). Under dark-adapted conditions with bright flashes, to derive mixed rod and cone responses, a photoreceptor-mediated a-wave (negative deflection) followed by an inner retina-mediated b-wave (positive deflection) was recorded. The a-wave amplitude was not different (*p* = 0.12), but the b-wave amplitude was significantly reduced in PDGFRα KO mice (*p* < 0.01) (Figure 4D). Decreased b-wave amplitudes in the ERG are a sign of dysfunctional neuronal information processing in the inner retina. The timing of the response, the implicit time, was measured as the time between the flash and the amplitude peak. No differences were found in the ERG implicit time for rod-specific, cone-specific, or mixed b-wave, or in the ERG implicit time-mixed a-wave (Figure 4E–G). In addition, we analyzed the oscillatory potentials (OPs). OPs are high-frequency wavelets on the ascending slope of the b-wave that reflect inner retinal function [31]. The OP amplitudes and the OP implicit times were not significantly different between PDGFRα KO and wildtype mice (Figure 4H–J). 

These findings imply that PDGF signaling in Müller cells has a direct influence on the overall retinal physiology.

### 2.4. Vascular Effects of Müller Cell-Specific PDGFRα Knockout in Normal Retina and in a Model of Choroidal Neovascularization (CNV)

**Figure 5:**
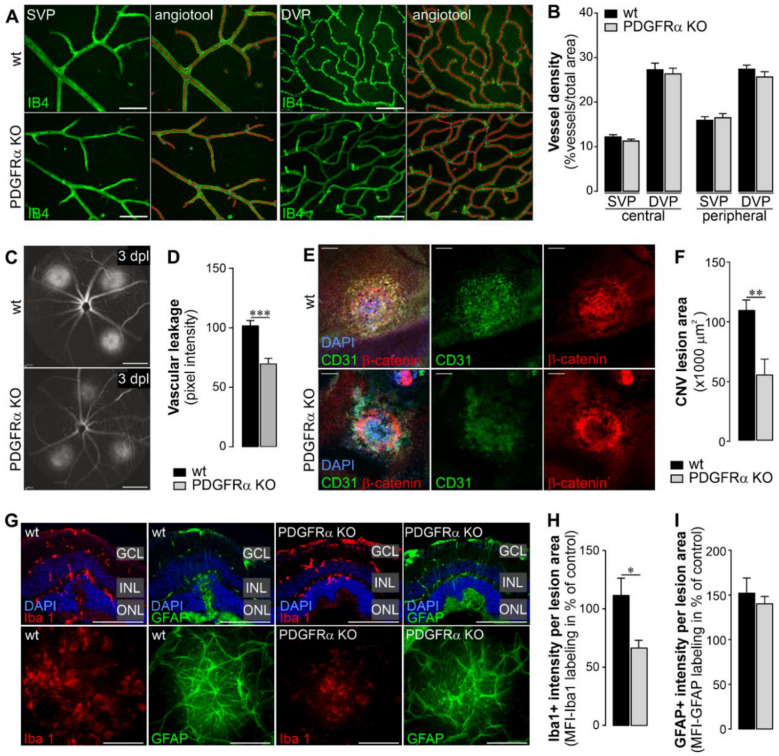
Vascular and inflammatory effects of specific deletion of PDGFRα in Müller cells. (**A**) Retinae from wildtype (wt) and PDGFRα KO mice were dissected, labeled with isolectin B4 (IB4) to label blood vessels, and flatmounted. Panels show representative retinal images from the superficial vascular plexus (SVP) and the deep vascular plexus (DVP) taken in central areas from the flatmount preparation. The resulting image after analysis of vessel density by the angiotool is shown exemplarily. Scale bars, 100 µm. (**B**) Quantification of vessel density was performed by analyzing confocal optical slices taken from the SVP and the DVP (composed of the inner deep vascular plexus and the outer deep vascular plexus, respectively) in central and peripheral areas of the retina from wt and PDGFRα KO mice. Bars represent mean ± SEM from 3–5 animals. (**C**) Fluorescence angiography performed on eyes at 3 days post laser lesion (dpl) to analyze the functional integrity of the retinal/choroidal blood vessels in wt and PDGFRα KO mice. Scale bars, 200 µm. (**D**) Quantification of vascular leakage by analyzing pixel intensities at 3 days after laser-induced retinal damage. Data are shown as mean ± SEM (n = 6 eyes). (**E**) Representative micrographs from laser lesions of retinal eye cups after careful removal of the retina from wt and PDGFRα KO mice scanned via confocal microscopy. Endothelial cells were delineated by CD31-immunoreactivity and RPE cells were detected using β-catenin-labeling. DAPI was used for nuclear staining. Scale bars, 100 µm. (**F**) The area of the lesioned RPE/choroid in flatmount preparations was measured on the basis of CD31 labeling. Data are shown as mean ± SEM (n = 4–5). (**G**) Representative micrographs from laser lesions from wt and PDGFRα KO retinae scanned via confocal microscopy. Microglia/macrophages were delineated by Iba1-immunoreactivity and reactive Müller cells as well as astrocytes in the nerve fiber layer were highlighted by staining GFAP. *Top*, retinal sections through laser lesions. Nuclei were counterstained with DAPI. GCL, ganglion cell layer; INL, inner nuclear layer; ONL, outer nuclear layer. *Bottom*, maximum projection of z-scans across a laser lesion performed on retinal flatmount preparations. Scale bars, 100 µm. (**H**) The labeling intensity for Iba1 (microglia/macrophage marker) was measured across the whole scan field centered over the lesioned retinal area in flatmount preparations. (**I**) The labeling intensity for GFAP (marker for astrocytes and reactive Müller glia) was measured across the whole scan field centered over the lesioned retinal area in flatmount preparations (**H**,**I**). Bars represent mean ± SEM (n = 5). (**B**,**D**,**F**,**H**,**I**) Mann–Whitney U-test; * *p* < 0.05, ** *p* < 0.001, *** *p* < 0.001.

An intact retinal microvasculature is crucial to maintain an adequate blood supply and the functional capacity of the neuronal circuits in the retina. Therefore, we evaluated whether the microvasculature in the PDGFRα KO mouse showed any abnormalities. For this purpose, the endothelium of blood vessels in retinal flatmounts was stained with isolectin B4 (Figure 5A). The superficial vascular plexus was distinguished from the deep vascular plexus (SVP and DVP, respectively) of retinae from wildtype and PDGFRα KO mice in central and peripheral areas of respective specimens. Implementing the angiotool analysis as described by Zudaire et al. [32], we did not detect any major differences between the vasculature from wildtype and PDGFRα KO mice (Figure 5A). Additionally, there was no significant difference regarding vessel density between wildtype and PDGFRα KO retinae when analyzing the SVP or DVP from central or peripheral areas (Figure 5B). 

Given that targeting PDGF has recently been tested as novel therapeutic strategy to treat detrimental neovascularization [8], we continued to analyze whether Müller cell-specific deletion of PDGFRα affected the vascular response to noxious stimuli such as laser lesions of the outer retina, underlying RPE and Bruch’s membrane and mimicking aspects of wet age-related macular degeneration [33]. In comparison to their wildtype littermates, Müller cell-specific PDGFRα KO mice presented with a reduced vascular leakage in the CNV model at three days after laser lesions were in induced (Figure 5C,D). Of note, the area of tissue damage at the level of the RPE/choroid in response to the laser application was also statistically smaller in knockout animals (Figure 5E,F). This suggests that blocking PDGF signaling specifically in Müller cells is sufficient to elicit potent beneficial vascular effects. 

Next, we investigated the infiltration of macrophages/microglia and Müller cell gliosis at the lesion site in overlaying retinal tissue. The microglial response in the KO animals was significantly decreased in comparison to that in wildtype littermates (Figure 5G,H). In contrast, Müller cell gliosis measured on basis of GFAP upregulation did not differ between the two genotypes (Figure 5G,I).

### 2.5. Pharmacological Inhibition of PDGF Signaling Had Similar Effects as the Müller Cell-Specific PDGFRα Knockout in the CNV Model

**Figure 6:**
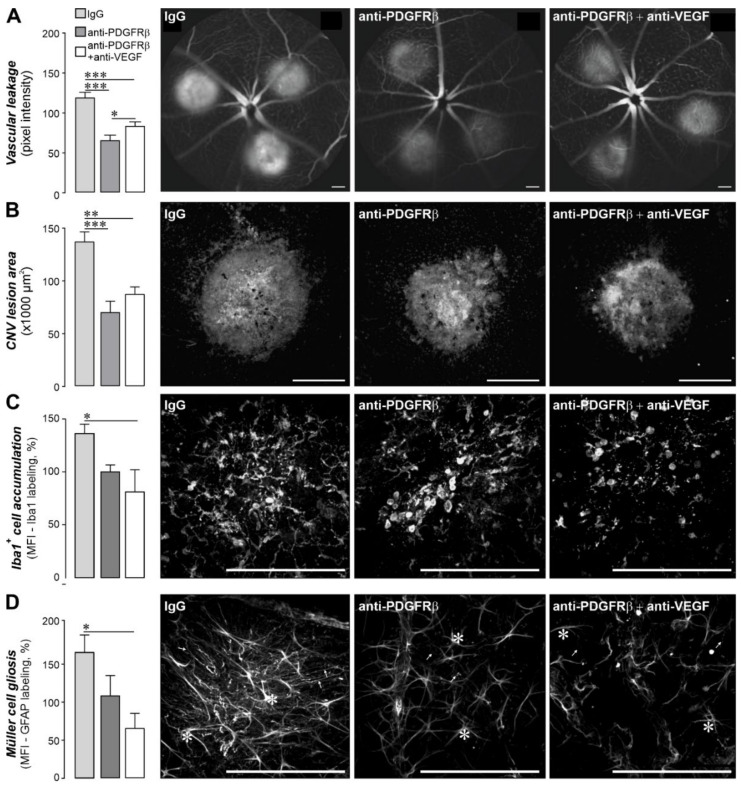
Anti-PDGFRβ treatment has similar effects on vascular integrity and the glial response pattern as a combined anti-PDGFRβ/anti-VEGF therapy in a murine model of choroidal neovascularization (CNV). (**A**) *Left*, quantification of vascular leakage by analyzing pixel intensities at 3 days after laser-induced retinal damage. The follow antibody concentrations were used: IgG control (1 µg), anti-PDGFRβ (1 µg), or anti-PDGFRβ + anti-VEGF (each 1 µg). Data are shown as mean ± SEM (n = 14–20 eyes). *Right*, fluorescence angiography performed on eyes at 3 days after laser lesion to assess data on the functional integrity of the retinal/choroidal blood vessels with or without treatment with anti-PDGFRβ alone or in combination with anti-VEGF antibodies. (**B**) *Left*, the area of the lesioned RPE/choroid in flatmount preparations was measured on basis of the CD31 labeling. Data are shown as mean ± SEM (n = 6–10). *Right*, representative micrographs from laser lesions from eyes that either received control IgG, anti-PDGFRβ alone, or in combination with anti-VEGF antibodies scanned via confocal microscopy. Endothelial cells are delineated by CD31 immunoreactivity. (**C**) *Left*, the labeling intensity for Iba1 (microglia/macrophage marker) was measured across the whole scan field (450 µm × 450 µm) centered over the lesioned area in retinal flatmount preparations. A maximum intensity projection from a z-stack scanned through whole thickness of that retinal area was used for evaluation. Data are shown as mean ± SEM (n = 4 animals per treatment group). *Right*, representative maximum intensity projection of z-stacks from Iba1-stained retinal flatmount preparations collected from mice from the respective treatment groups scanned via confocal microscopy. (**D**) *Left*, the labeling intensity for GFAP (marker for retinal astrocytes (asterisk) and gliotic Müller cells (arrows)) was measured across the whole scan field (450 µm × 450 µm) centered over the lesioned retinal area in flatmount preparations. A maximum intensity projection from a z-stack scanned through whole thickness of that retinal area was used for evaluation. Data are shown as mean ± SEM (n = 4 animals per treatment group). *Right*, representative maximum intensity projection of z-stacks from GFAP-stained retinal flatmount preparations collected from mice from the respective treatment groups scanned via confocal microscopy. (**A**–**D**) Mann–Whitney U-test, * *p* < 0.05, ** *p* < 0.01, *** *p* < 0.001. (**A**–**D**) Scale bars, 200 µm.

The findings that neovascular alterations are less pronounced in Müller cell-specific PDGFRα mice (Figure 5) prompted us to ask whether pharmacological inhibition of PDGF signaling has effects comparable to its genetic ablation. Similar to the experiments in the PDGFRα knockout mice, eyes were lasered in three spots and immediately thereafter were injected (i) either with control IgG, (ii) anti-PDGFRβ antibody (known to effectively block PDGF signaling) alone, or (iii) in combination with an anti-VEGF antibody. The animals were analyzed according to the same protocols used for the Müller cell-specific PDGFRα KO mice. Fluorescence angiography three days after laser lesion demonstrated that anti-PDGFRβ treatment alone showed similar beneficial effects regarding vascular leakage and lesion size as in combination with anti-VEGF (Figure 6A,B) and as found in Müller cell-specific PDGFRα mice (Figure 5C–F). This suggests that the blockade of PDGFR signaling alone (no matter whether it is targeted via PDGFRα or -β) is sufficient to achieve a significantly improved vascular integrity (Figure 5D and Figure 6A) and smaller lesion size at the level of the RPE/choroid (Figure 5F and Figure 6B) compared to lasered eyes treated with the control IgG or in respective wildtypes. Of note, no clear additive effect of a concomitant anti-VEGF treatment was observed in this experimental setting.

Analogous to the experiments in the Müller cell-specific PDGFRα KO mice, less microglia accumulated in the retina tissue at the lesion site if mice were treated either with anti-PDGFRα alone or in combination with anti-VEGF antibodies (Figure 6C). The effect size was comparable to what we found in PDGFRα KO mice (Figure 5H). Moreover, we found strong upregulation of the Müller cell gliosis marker GFAP in the lesioned area (Figure 6D), but less Müller cells up-regulated GFAP in retinae if treated with anti-PDGFRβ or if the combinatorial approach was applied (Figure 6D). In sum, the analysis of marker gene expression confirms an expected glial response upon tissue damage, but only the combination of PDGFRβ and anti-VEGF antibodies seems to significantly dampen Müller gliosis induction. 

## 3. Discussion

PDGF signaling has been shown to be essential for embryonic development of the retina [34] and a contribution to pathological ocular neovascularization is well established [35]. However, in the mature retina the physiological function of PDGF signaling remains largely unexplored. PDGFRα expression is confined to a few cell types in the eye, such as astrocytes and amacrine cells [36]. Others reported its expression in Müller glia in the developing [11] and adult rodent retina [12,37]. Supporting evidence for PDGFRα expression in Müller glia comes from studies using MIO-M1 cells, an immortalized human Müller cell line [38], and rat primary Müller cell cultures [11]. However, to date an unequivocal proof of PDGFRα expression by Müller cells and data about its functional relevance are lacking. Using a novel technique to purify intact Müller cells from adult mouse retinas [26], we confirmed that they indeed express *pdgfra* at the mRNA and protein level and, importantly, that expression in Müller cells is higher than in any other retinal cell type (Figure 1C). Moreover, we found other components of the PDGF pathway such as *pdgfrb* and the ligand *pdgfb* to be expressed at considerable levels by Müller cells (Figure 1C), suggesting a key role of PDGF signaling for Müller cell physiology. 

Knockout studies of PDGF family genes have demonstrated that they play an essential role during development and specifically on the establishment of the neurovascular unit. A mutation of PDGFRα in a domain key to mediate downstream signal transduction resulted in defects in the blood and kidney glomerulus formation so that homozygotes died perinatally [39]. In line with that, a null mutation in the gene encoding the PDGF-BB chain led to lethal renal and hematological abnormalities plus cardiovascular defects [40]. Given the perinatal death of the respective KO animals, many primary functions of PDGF signaling remained elusive until evaluated in experiments using conditional gene ablation as in the current study. In the retina, the importance of PDGF-BB has been shown by the ablation of endothelium-derived PDGF-BB, which resulted in reduced retinal pericyte coverage. These mice presented with a vascular phenotype recapitulating features of diabetic retinopathy (venous tortuosity, regressing capillary branches, and microaneurysms) [41]. A similar effect was found in a CNS-specific PDGFR α mutant mouse [42]. These animals exhibited vascular leakage and retinal neurodegeneration, including ganglion cell apoptosis. Finally, it has been described that when PDGF signaling was disrupted in the developing eye by injection of anti-PDGF-A antibodies or the extracellular domain for PDGFRα, a strong growth inhibition of the retinal astrocyte network and a concomitantly reduced vascular plexus formation was induced [43]. Despite these reports, we did not find major alterations in retinal morphology, vascular architecture, or signs of cell death (Figure 2; Figures S1 and S2) in the newly generated Müller cell-specific PDGFRα KO mouse model. It is likely that the cell type (Müller glia) and time-specific knockout of PDGFRα accounts for the lack of a morphological phenotype. The critical phases of migration and maturation of retinal cells and the establishment of the retinal vascular plexus is largely finished by the end of the fourth postnatal week in mice [44,45,46] and, thus, weeks before the actual knockout was induced by tamoxifen injection (adult mice, 12–16 weeks of age). 

Intense neural activity leads to continuous, local changes of osmotic gradients and ion concentrations. Müller cells are prominently involved in controlling the extracellular ion and fluid homeostasis in the retina and especially of its inner layers (GCL, INL) [47]. Growth factors such as VEGF or heparin-binding epidermal growth factor (HB-EGF), acting via receptor tyrosine kinase receptors such as PDGF, induce a glutamatergic–purinergic signaling cascade key for Müller cell volume regulation and, therefore, also their homeostasis functions [27,48]. In line with that, we found that the capability of PDGFRα-deficient Müller cells to counterbalance osmotic stress was severely diminished (Figure 3D). Finding no major morphological differences together with this clear functional phenotype seems counterintuitive. However, this can possibly be explained by the fact that under normal conditions the remaining homeostatic function of Müller cells, e.g., mediated by Kir4.1 potassium channels, showed to be normally expressed in the KO mice, and this seems to be sufficient to support neuronal survival. Of note, in a recent study we observed a similar phenotype in mice deficient in the ATP receptor P2Y_1_. As in Müller cell-specific PDGFRα KO mice, Müller cells of P2Y_1_ KO mice were unable to perform efficient cell volume regulation, but no effects on retinal integrity were observed [49]. 

Having demonstrated Müller cell dysfunction in PDGFRα KO prompted the question of whether visual function might be compromised, given earlier findings that genetically or pharmacological interference with Müller cell homeostasis functions led to changes in the retinal light response. A pharmacological blockade of the glutamate clearance from the synaptic cleft by Müller cells resulted in a reduced b-wave amplitude [50]. Similarly, knockout of the water channel AQP4, also key for Müller cell volume regulation [51], led to alterations of the b-wave in respective mice [52]. In line with this, a recent study on neuromyelitis optica, an autoimmune disease where anti-AQP4 antibodies are formed [53], correlated Müller cell dysfunction with electrophysiological disturbances in affected patients. In sum, these studies point out the importance of Müller glia homeostasis in regulating neuronal function and, thus, the retinal light response. This idea is further supported by a recent study, where the accumulation of N-(3-formyl-3,4-dehydropiperidino)lysine (FDP)-lysine in Müller glial was inhibited using a targeted Müller glia acrolein-scavenging drug to improve glial function in a rat model of diabetic retinopathy [54]. This treatment resulted in a significantly improved disease outcome, including an increased ERG responsiveness. Accordingly, we hypothesize that the dysfunctional Müller cell homeostasis in the PDGFRα KO mice contributes to the observed reduction in retinal light response measured by ERG recordings (Figure 4). Since significant differences were primarily observed for the b-wave together with the understanding that the b-wave reflects the activity of cells of the inner retinal layers, we speculate that especially cells of the inner retinal layers (INL, GCL) closely rely on intact Müller cells to integrate and process signals from photoreceptors normally. This would be in accordance with our earlier study demonstrating that ganglion and amacrine cells are more susceptible to a diminished homeostasis function of Müller cells [49]. 

Of note, the ERG findings seem to partially contradict results from the initial morphometric analysis where no major differences regarding gross retinal architecture were observed (Figure 2). However, it is well described that even if cells per se are still present, their function can be largely diminished, thus leading to early changes in ERG amplitudes [55].

The importance of functional Müller cells for the integrity of the retinal vascular system was demonstrated by Shen et al [17]. Selective Müller cell ablation led to vascular leakage and intraretinal neovascularization. In the absence of any specific exogenous challenge, the retinal microvasculature from Müller cell-specific PDGFRα KO mice showed no anomalies. The vascular density of the different plexus along the central and peripheral retina remained unchanged and we did not detect signs of degeneration of vascular cells, e.g., by evaluation via a TUNEL assay (Figure S2C). Although key for Müller cell volume regulation, this implies that PDGFRα in Müller cells is not essential to maintain mature retinal microvessels—at least not over the times span investigated in the present study. Interestingly, PDGFRα deficiency in Müller cells even turned out to mitigate vascular abnormalities in the CNV model used to test for the tissue response on a pathological stimulus (Figure 5). This model recapitulates aspects of neovascular age-related macular degeneration (AMD), is highly reproducible [56,57,58] and evaluating leakage of fluorescein dye by angiography is a clinically relevant method to analyze the vasopermeability of CNV lesions [59,60]. The reduced vascular hyperpermeability and growth of laser-induced CNV lesions in Müller cell-specific PDGFRα KO mice are key findings of the present study (Figure 5). These results are in line with a recent study demonstrating that inhibition of PDGF signaling using an aptamer in the same CNV model also reduced the size of the laser lesions [6]. Furthermore, systemic administration of imatinib (targeting PDGFRα and β) improved the outcome in a mouse model of oxygen-induced retinopathy—another model of retinal neovascularization [61].

In addition to the described vascular changes, the CNV model comprises inflammatory components possibly contributing to lesion formation. It has been shown that macrophages/microglia are involved in CNV formation in animal models and in AMD patients [62,63]. Interestingly, we found that the disruption of Müller cell-specific PDGF signaling impacts inflammation during pathological angiogenesis, as the recruitment of microglia and macrophages was significantly diminished (Figure 5G–I). Others achieved similar effects by blocking VEGF receptor 1 and 2 [64]. Two possible pathways why the vascular permeability, the lesion area, and the microglial response upon anti-VEGF treatment were reduced have been discussed: (i) The disruption of VEGF either disturbed an autocrine mechanism regulating the production of pro-inflammatory factors, or (ii) it blocked paracrine intercellular communication, stimulating neighboring cells to generate inflammatory factors. The precise mechanism underlying the beneficial effects observed on vascular hyperpermeability and inflammation in the Müller cell-specific PDGFRα KO mice remains to be identified, but since receptors and ligands are expressed by Müller cells and cells of the neurovascular unit, paracrine and autocrine pathways are likely to be involved.

Next, we compared effects observed in Müller cell-specific PDGFRα KO mice with the pharmacological inhibition of the PDGF axis alone and in combination with anti-VEGF antibodies. Anti-VEGF drugs have revolutionized the management of patients with retinal neovascular diseases [65]. Despite currently being the gold-standard treatment, anti-VEGF therapy is not universally effective [66,67]. There are patients that are unresponsive or become refractory [68,69]. In consequence, novel treatment approaches combining anti-VEGF therapy with other agents (including PDGF) acting synergistically are subject of (pre-)clinical trials [6,9]. Interestingly, the effectiveness of targeting the PDGF pathway in Müller cells either genetically or by pharmacological blockade (anti-PDGFRα antibodies alone or in combination with anti-VEGF antibodies) to reduce neovascularization and neuroinflammation was highly comparable in the CNV model, but did not show significant synergism as one might would have expected from earlier reports (Figure 5 and Figure 6). There are several factors potentially influencing the synergistic effects of a parallel VEGF and PDGF blockade. Among those the route of application, the assessment time points, and the animal model used should be considered. In comparison with Jo et al. [6], we analyzed the effects at a different time point—namely, at three days post laser lesion instead of 14 days. Our rationale was to avoid the potential spontaneous regression of the vascular lesion that was reported by others [70]. Possibly the inhibitory effect of the combinatorial treatment or the PDGFRα KO on microglial and Müller cell activation holds potential to prevent excessive scar formation, which should be characterized in depth in future studies analyzing later time points (e.g., at two and three weeks after laser lesion). This would then also help to approach the question of potential synergistic effects at later time points in more detail.

Finally, it needs to be discussed whether a pharmacological blockade of retinal PDGF signaling does result in similar adverse effects on Müller cell and retinal function, as described for the PDGFRα KO mice. For sure, here the main question is the time span over which the therapy is implemented and the efficiency of the blockade of the PDGF pathway over time. If the therapeutic regimen follows the current scheme of monthly or even less frequent intravitreal antibody injections, it can be assumed that PDGF signaling will not be totally blocked throughout the complete treatment interval. This could, on the one hand, limit the therapeutic effect on neovessels, but on the other be beneficial to maintain a basal Müller cell homeostasis function that is key for normal neuronal function, which we demonstrated here. In the case of gene therapeutic approaches, e.g., expressing the therapeutic antibody via adeno-associated virus (AAV)-based vectors and as currently tested in clinical trials targeting the VEGF pathway ([71,72]; reviewed in [73]), we would speculate that unwarranted side effects on Müller cell homeostatic functions would be more pronounced. This hypothesis is supported be findings from VEGF knockout mice, demonstrating that total absence thereof leads to neurodegeneration [10]. Together with the data from our present study, we consider that these aspects have to be taken into account when determining the safety profile of such new treatment approaches of neovascular diseases permanently blocking retinal VEGF or PDGF signaling pathways. 

## 4. Materials and Methods

### 4.1. Experimental Animals

Mice were maintained with ad libitum water and food access in an air-conditioned room on a 12 h light–dark schedule. Experiments were done in accordance with the ARVO Statement for the Use of Animals in Ophthalmic and Vision Research and with the European Community Council Directive 2010/63/EU. They were approved by the local authorities (55.2-2532.Vet_02_18_47; Bavaria, Germany and 84-02.04.2017.A130; Landesamt für Natur, Umwelt und Verbraucherschutz Nordrhein-Westfalen, Germany). All experimental procedures were performed with adult (2–6-month-old) animals from mixed genders of respective mouse strains. For inducible deletion of PDGFRα in Müller cells, mice carrying floxed alleles (PDGFRα^fl/fl^) were crossed with Glast-CreER^T2^ mice [74]. Mice were bred from heterozygous Glast-CreERT2 and PDGFRα^+/fl^ parents to obtain Glast-CreERT2; PDGFRα^fl/fl^ homozygotes and Glast-CreERT2; PDGFRα^+/+^ littermate wildtype controls, respectively. To revalidate the efficiency and specificity to Müller cells of the Glast-CreER^T2^ mice, they were also crossbred with the tdTomato-expressing reporter mouse line Ai3 [24].

### 4.2. Recombination Induced by Tamoxifen Administration

To induce Cre-mediated recombination, tamoxifen was thoroughly dissolved at 42 °C in ethanol (100 mg/mL; Sigma Aldrich, Schnelldorf, Germany), diluted with corn oil to a final concentration of 10 mg/mL, and finally administered intraperitoneally (20 μg/g body weight) every day for 3 consecutive days. Mice were analyzed 4 weeks after the final day of tamoxifen administration to ensure clearance of the targeted protein.

### 4.3. In Vivo Laser Model for Choroidal Neovascularization (CNV)

Mice were anesthetized by intraperitoneal injection of a mixture of ketamine/xylazine (100 mg/5 mg per kg bodyweight), and laser coagulation of the retina was performed using a 532 nm green laser with a slit-lamp mounted diode laser system (Quantel Medical Vitra, Cournon d’Auvergne, France). The pupil dilation was achieved with 0.5% tropicamide-phenylephrine 2.5% eye drops before laser treatment. Three laser burns per eye (energy 125 mW, duration 100 ms, spot size 100 µm) were applied around the optic nerve of both eyes with a cover glass as contact lens for fundus fluorescein angiography (FA) and immunohistochemistry (IHC) [75]. For mRNA expression analysis the number of laser spots per eye was 20. Rupture of the Bruch’s membrane was validated post-laser by examination of retinal structure in vivo using a Spectralis^TM^ HRA/OCT device (Heidelberg Engineering, Heidelberg, Germany). Lesions with hemorrhage and eye opacities with a pre-existing corneal scar or cataract as well as insufficient disruption of the Bruch’s membrane were excluded from analysis.

### 4.4. Intravitreal Drug Administration 

The animals were randomly assigned to the experimental groups. The compounds used in this study were injected intravitreally (all diluted in 1x phosphate buffered saline) immediately after inducing CNV: 1 μL of either anti-PDGFRβ (1 μg, CD140b (APB5), 16-1402-82 eBioscience; San Diego, CA, USA), anti-VEGF-A (VEGF_164_, AF-493-NA, R&D Systems, Wiesbaden, Germany), and combined anti-PDGFRβ (each 1 μg) or IgG2a kappa isotype control (1 μg, 16-4321-81, eBioscience). To intravitreally administer the aforementioned reagents, a 34-gauge needle was inserted into the vitreal cavity approximately 1.5 mm below the limbus using a NanoFil Syringe (WorldPrecision Instruments, Sarasota, FL, USA) and the compounds injected bilaterally.

### 4.5. Fluorescein Angiography (FA)

After the induction of anesthesia and pupil dilation of the animals, the vascular leakage was analyzed with the FA mode of the Spectralis^TM^ HRA/OCT device. For this 100 µL of 2.5% fluorescein (Alcon, Großwallstadt, Germany) diluted in 0.9% sodium chloride was administered intraperitoneally. Images were acquired 10 min after fluorescein administration (late phase). Vascular leakage at the CNV area was analyzed by quantifying the pixel intensity in two regions of interest (ROI) within and one ROI outside each laser spot using the program ImageJ (National Institutes of Health (NIH), Bethesda, MD, USA), and then the background pixel intensity was subtracted from the laser spot values. All 3 laser lesions were averaged to give one experimental value per eye. 

### 4.6. Immunohistochemical Analysis of Retinal Tissue and Image Analysis

For immunohistochemical studies, mouse eyes were enucleated and immediately fixed in 4% paraformaldehyde (Carl Roth, Karlsruhe, Germany) in pH 7.4 for 2 h at room temperature. The anterior segments were carefully dissected out under a biopsy microscope, and the neurosensory retina and the RPE-choroid-sclera were detached and isolated from the optic nerve head. Four to six radial cuts in the remaining retina and RPE-choroid-sclera were made in the direction of the equator. The dissected retinal and RPE-choroid-sclera flatmounts were incubated in the primary antibodies diluted in blocking solution (3% dimethyl sulfoxide, 0.3% Triton X-100, and 5% of an appropriate serum diluted in PBS) overnight at 4 °C (Table S1). The flatmounts were then incubated in a 1:1000 dilution of their respective fluorescent secondary antibodies overnight at 4 °C, followed by washing with PBS.

Another group of mice was euthanized and their eye cups were dissected and fixed in 4% paraformaldehyde solution for 2 h and cryoprotected in a solution of 30% sucrose overnight. Then they were embedded in cryomatrix^TM^ (Thermo Fisher Scientific, Schwerte, Germany) and sectioned into 10 μm-thick slices and stained using a similar protocol as for retinal flatmounts. Experiments in which primary antibodies were omitted served as negative controls. 

After the final wash with 1X PBS, the retina, RPE-choroid flatmounts, and cryosections were mounted and embedded using aqua-poly/mount (18606-20; Polysciences Europe, Hirschberg an der Bergstrasse, Germany). Samples were scanned using confocal microscopy (custom-made VisiScope CSU-X1 confocal system equipped with a high-resolution sCMOS camera; Visitron Systems, Puchheim, Germany). The apoptosis of retinal cells was assayed using a terminal deoxynucleotidyl transferase dUTP Nick End Labeling (TUNEL) assay according to the manufacture’s specifications (Roche Molecular Biochemicals, Mannheim, Germany). Using ImageJ [76], we quantified the cell nuclei in the different retinal layers and calretinin-positive cells. Areas of the central retina that were 150 μm wide and close to the optic nerve head (to avoid eccentricity-dependent effects) were chosen for the analysis (optical slice thickness, 1.5 μm). For histological analysis, at least 3 animals per genotype were investigated, from which representative micrographs were chosen for presentation in respective figures.

### 4.7. Retinal Vasculature Labeling and Quantification

Retinal flatmounts were prepared and the blood vessels were stained using fluorescein-conjugated isolectin B4 (IB4) (Table S1). Immunostaining with IB4 and DAPI was performed for two days at 4 °C. Following incubation with the antibodies, retinae were washed 3 times for 5 min each in phosphate buffer solution (PBS) and were embedded using aqua-poly/mount (18606-20; Polysciences Europe). For analysis, multiplane z-series were collected from fields of views of the vascular network from wildtype and KO mice by using 25× objective lens (VisiScope CSU-X1 confocal system; Visitron Systems). The series included the vitreal surface to the outer plexiform layer (OPL), and we captured at least 4 fluorescent images/retina/central and peripheral areas. Next, we performed a z-stack projection, dividing the z-series in two sets: the superficial vascular plexus (SVP) and the deep vascular plexus (DVP). The retinal vessel density was quantified using the angiotool software [32].

### 4.8. CNV Lesion Area Quantification

To analyze the lesion area, the RPE-choroid-sclera complex was stained using two different markers to delimitate the laser injury site—the endothelial cell marker CD31 and the RPE marker β-catenin (evaluation of RPE cell disruption). Images were obtained using a 10X objective lens focusing on the laser spot by confocal microscopy (VisiScope CSU-X1 confocal system, Visitron System). ImageJ (NIH) was used to measure the lesion size in a masked fashion. Lesions were excluded when there was obvious damage due to tissue processing. The mean lesion size obtained from all 3 lesions per eye was plotted as one experimental value per animal and was subjected to further statistical analysis.

### 4.9. Müller Cell Reactivity and Macrophage/Microglia Accumulation Evaluation

Müller cell reactivity and macrophage/microglia recruitment were evaluated by measuring the intensity of the GFAP+ and Iba+ signal respectively in the retinal flatmounts. For that purpose, z-stacks reaching 100–120 μm into the retinal tissue (starting at the nerve fiber layer) were obtained using a 25× objective lens of the confocal microscope (previously described) at the site of the lesion. The mean fluorescence intensity was analyzed by ImageJ software (NIH). The final values are expressed as percentages, after background signal normalization to that detected in non-lesioned regions. 

### 4.10. Müller Cell Soma Swelling and Volume Regulation

Freshly isolated retinal slices (1 mm thickness) were prepared and placed with the photoreceptor side onto membrane filters (0.45 μm, diameter 50 mm; GE Healthcare Life Sciences, Freiburg, Germany). Volume changes of Müller cell somata evoked under isotonic conditions and after hypoosmotic challenge (60% of control osmolarity) were measured in the inner nuclear layer of retinal slices as previously described [77]. The retinal slices from wildtype and KO mice were placed in a perfusion chamber (custom made) and loaded with the vital dye Mitotracker Orange (10 μM, excitation 543 nm, emission 560 nm-long pass filter; Thermo Fisher Scientific). The Mitotracker Orange dye selectively stained Müller glia [78], and the stock solution was prepared in DMSO and diluted 1:1000 in PBS. The slices were examined during exposure to a hypotonic solution for 4 min with or without test agents using confocal microscopy (custom-made VisiScope CSU-X1 confocal system equipped with a high-resolution sCMOS camera; Visitron Systems). The cell soma area of labeled Müller cells was cross-sectionally measured (ImageJ). The blocking agent was pre-incubated for 10 min in extracellular solution; AC710 (PDGFR family inhibitor; Tocris Bioscience, Bristol, UK) 100 nM. PDGF-BB, a PDGF receptor α/β agonist (R&D Systems), was applied simultaneously with the hypotonic solution.

### 4.11. Magnetic-Activated Cell Sorting (MACS) of Retinal Cell Types

Müller cells were enriched from isolated retinae by magnetic-activated cell sorting (MACS) as described previously [25]. Briefly, the isolated retinae were digested in papain (0.2 mg/mL) for 30 min at 37 °C, in Ca^2+^- and Mg^2+^-free extracellular solution. Afterwards, the retinae were shortly incubated with DNaseI (200 U/mL, Roche Molecular Biochemicals) and triturated. After centrifugation, the retinal tissue was resuspended and subsequently incubated with CD11b and CD31 microbeads according to the standard manufacturer’s protocol (Miltenyi Biotec, Bergisch Gladbach, Germany) to obtain microglial and vascular cell-enriched fractions; the respective binding cells were depleted from the retinal suspension using large cell columns (Miltenyi Biotec) prior to Müller cell enrichment. After washing, Müller cells were purified by incubating the retinal suspension in extracellular solution containing biotinylated anti-CD29 (Miltenyi Biotec) for 15 min at 4 °C. The cell population in the flow through depleted CD11b+, CD31+, and CD29+ cells was considered the neuronal fraction [26,79]). Nonetheless, a minor contamination with other cell types could not be ruled out. To assess the purity of the CD29+ Müller cell fraction, the cell suspension was checked via immunostaining using the Müller glia marker glutamine synthetase together with a DAPI counterstain to quantify total cell numbers. As described previously [25], Müller cell purity was more than 80%, with photoreceptors being the main contaminating cell type. Freshly isolated CD29+ Müller cells and the respective CD29- neuronal fraction from PDGFRα KO and wildtype mice were used to validate the Müller cell-specific deletion of PDGFRα by immunofluorescence using the previously described protocol for cryosection staining.

### 4.12. Gene Expression Analyses

Total RNA was isolated after the immunoseparation of retinal cell subpopulations using the RNeasy Micro Kit (Purelink RNA Micro Scale kit, Thermo Fisher Scientific) with the supplied manufacturer’s instructions. To remove genomic DNA contamination a DNase digestion step was added (Roche Molecular Biochemicals). cDNA was synthetized with the RevertAid H Minus First-strand cDNA Synthesis kit (Fermentas by Thermo Fisher Scientific, Schwerte, Germany) using 10–50 ng of total RNA. To evaluate candidate gene expression levels, primers were designed using the Universal ProbeLibrary Assay Design Center (Roche Molecular Biochemicals). Quantitative real-time PCR (qPCR) was performed using cDNA from respective enriched cell populations with the TaqMan hPSC Scorecard^TM^ Panel (384 well, ViiA7, Life Technologies, Darmstadt, Germany) according to the company’s guidelines. Expression levels of respective complement genes (for primer sequences see Table 1) were normalized to the expression of isocitrate dehydrogenase 3 (NAD+) beta (idh3b) that we validated in a recent studied to fulfill the criteria of a good housekeeping gene across retinal cell populations [80].

### 4.13. Electroretinogram Recording

Electroretinogram (ERG) recordings were performed 4 weeks after tamoxifen injection and compared to age-matched wildtype littermates. The mice were dark adapted overnight, anesthetized by intraperitoneal injection, and their pupils were fully dilated with 1% tropicamide and 10% phenylephrine. Recordings were carried out using an Espion ERG Diagnosys System (Dyagnosys LLL, Littleton, MA, USA) under dim red light illumination. During the recording and recovering, body temperature was maintained at 37 °C using a heating pad. Electrodes were placed on the cornea and ERG responses were simultaneously recorded from both eyes. Scotopic rod and mixed rod–cone responses were measured using light pulses of 0.001 and 3 cd × s/m^2^ (white 65,000 K), respectively. After 10 min of light adaptation in the Ganzfeld dome, photopic responses were recorded. Using light pulses of 30 cd × s/m^2^ (xenon), the a-wave amplitude was measured from baseline to the trough of the a-wave, and the b-wave amplitude from the trough of the a-wave to the peak of the b-wave. The a- and b-wave implicit times were quantified from the stimulus onset to the peak of the respective wave form. Additionally, the oscillatory potentials (OP) of the ERG were isolated and the first, second, and third positive peak amplitudes and implicit times were then plotted as OP1, OP2, and OP3, respectively.

### 4.14. Statistical Analysis

The data were analyzed using Prism (Graphpad Prism Software 7.1, San Diego, CA, USA) and reported as mean values ± standard error (SEM). In order to comply with the three Rs principle of animal welfare, the majority of the experiments in the current study came from 4 biological replicates unless stated otherwise. Therefore, we analyzed the significance levels using the non-parametric, two-tailed Mann–Whitney U-test, given that with this number of cases a normal Gaussian distribution could not be assumed. In each figure legend the specific n-values and significance levels are shown.

## 5. Conclusions

In summary, our data not only provide a first insight into the contribution of glial PDGF signaling to normal retinal function in general and for the physiology of Müller cells specifically, but we could also confirm that components of the PDGF pathway might serve as a target to block pathological ocular angiogenesis. However, our findings may also explain why recent clinical trials blocking PDGF signaling in conjunction with that of VEGF in neovascular retinal disease did not prove to be superior to anti-VEGF mono-therapy. Given that both VEGF and PDGF stimulate important homeostasis functions in Müller cells, too-efficient shutdown of these two pathways, specifically in case of the combinatorial treatment approach, may accelerate negative unwarranted side effects on the ion and fluid retinal homeostasis, interfering with the actual treatment goals, as seen in the failed Fovista phase III clinical trials (https://clinicaltrials.gov/ identifiers: NCT01944839, NCT01940900, NCT01940887; [81]).

## Figures and Tables

**Table 1:** Primer and TaqMan probe combinations for detection of the respective mRNA via qPCR.

Gene ID	Primer Sequences: Forward	Primer Sequences: Reverse	TaqMan^®^ Probe from Roche	Accession Number
idh3b	5′ gctgcggcatctcaatct 3′	5′ ccatgtctcgagtccgtacc 3′	# 67	NM_130884.4
aif1	5′ atctgccgtccaaacttga 3′	5′ ctaggtgggtcttgggaacc 3′	# 67	NM_001361501.1
glul	5′gcccaagtgtgtggaagag 3′	5′aaggggtctcgaaacatgg 3′	# 58	NM_008131.4
nrl	5′ tgcctttctggttctgacagt 3′	5′ gaaagccattctgggactga 3′	# 53	NM_008736.3
pecam1	5′ gctggtgctctatgcaagc 3′	5′ atggatgctgttgatggtga 3′	# 64	NM_008816.3
pdgfra	5′ cagacattgaccctgttcca 3′	5′ tctcttccgaagtctgtgagc 3′	# 69	NM_001083316.2
pdgfrb	5′ tcaagctgcaggtcaatgtc 3′	5′ ccattggcagggtgactc 3′	# 67	NM_001146268.1
pdgfb	5′ cggcctgtgactagaagtcc 3′	5′ gagcttgaggcgtcttgg 3′	# 32	NM_011057.4

## Data Availability

The data presented in this study are available on request from the corresponding author.

## Supplementary Materials

The following are available online at https://www.mdpi.com/1422-0067/22/3/1174/s1, Figure S1: Evaluation of PDGFRα expression in retinal sections of Müller cell-specific PDGFRα KO mice, Figure S2: Immunoreactivity of additional cell markers in Müller cell-specific PDGFRα retinae, Table S1: Information on primary and secondary antibodies.

## Abbreviations

AQP4	aquaporin 4
CNS	central nervous system
CNV	choroidal neovascularization
ERG	electroretinogram
GCL	ganglion cell layer
IPL	inner plexiform layer
INL	inner nuclear layer
OPL	outer plexiform layer
ONL	outer nuclear layer
PDGF	platelet-derived growth factor
PDGFR	platelet-derived growth factor receptor
PDGFRα KO	Müller cell-specific platelet-derived growth factor receptor alpha knockout
RPE	retinal pigment epithelium
TUNEL	terminal deoxynucleotidyl transferase dUTP nick end labeling
VEGF	vascular endothelial growth factor
wt	wildtype
