# Supplementary material for: PDGF Receptor Alpha Signaling Is Key for Müller Cell Homeostasis Functions
Source: Int J Mol Sci. 2021 Jan 25;22(3):1174. doi: 10.3390/ijms22031174 (PMC7865899; doi:10.3390/ijms22031174)
Supplement: Supplementary file 1 [file ijms-22-01174-s001.pdf]

Supplementary Materials:

Supplementary Table 1. Information on primary and secondary antibodies

**Primary antibodies**	**Host**	**Dilution**	**Source**	**Catalogue no.**
AQP4	Rabbit	1:500	Sigma	A5971
β-catenin	Rabbit	1:100	Cell Signaling	8480
CD31	Rat	1:200	BD Biosciences	550274
Calretinin	Goat	1:300	Swant	CG1
Cone-Arrestin	Rabbit	1:500	Merck	AB15282
GFAP	Moue	1:500	Sigma	G3893
Glutamine Synthetase (Glul)	Mouse	1:500	Millipore	MAB302
Iba1	Rabbit	1:500	Wako	019-19741
Kir4.1	Rabbit	1:400	Alomone labs	APC-035
PDE6b	Rabbit	1:2000	Thermofisher	PAI-722
PDGFRα	Rabbit	1:200	Cell Signaling	3164
PDGFRβ	Goat	1:200	R&D	AF1042
PKCα	Mouse	1:500	Santa Cruz	SC166350
SCGN	Rabbit	1:5000	Gift from Dr Ludwig Wagner, University of Vienna, Austria	
**Secondary antibodies**				
Alexa-Fluor 488	Donkey anti-rat	1:1000	LifeTech	A21208
Alexa-Fluor 647	Donkey anti-goat	1:1000	Dianova	705-605-003
Alexa-Fluor 555	Donkey anti-rabbit	1:1000	Invitrogen	A-31572
Alexa-Fluor 647	Goat anti-mouse	1:1000	Dianova	115-607-072
Cy3	Goat anti-rabbit	1:1000	Dianova	111-165-144
Cy5	Donkey anti-rabbit	1:1000	Dianova	711-175-152
Isolectin B4 FITC Conjugate		1:100	Sigma	L289

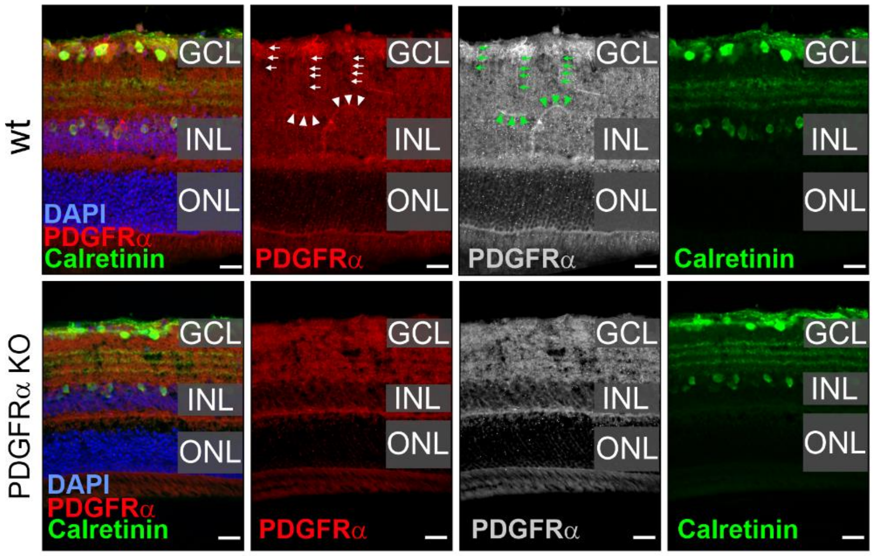

Figure S1. Evaluation of PDGFRα expression in retinal sections of Müller cell-specific PDGFRα KO mice. Retinae from wildtype (wt) and PDGFRα KO mice, were cross-sectioned and stained with antibodies for PDGFRα and calretinin (ganglion and amacrine cells). Vessels (arrow heads) and Müller glia (arrows) are immunoreactive for PDGFRα. Note the contrast in level of expression of PDGFRα in putative Müller cell processes in the wt retina in comparison with that in PDGFRα KO mice. Scale bars, 20 µm.

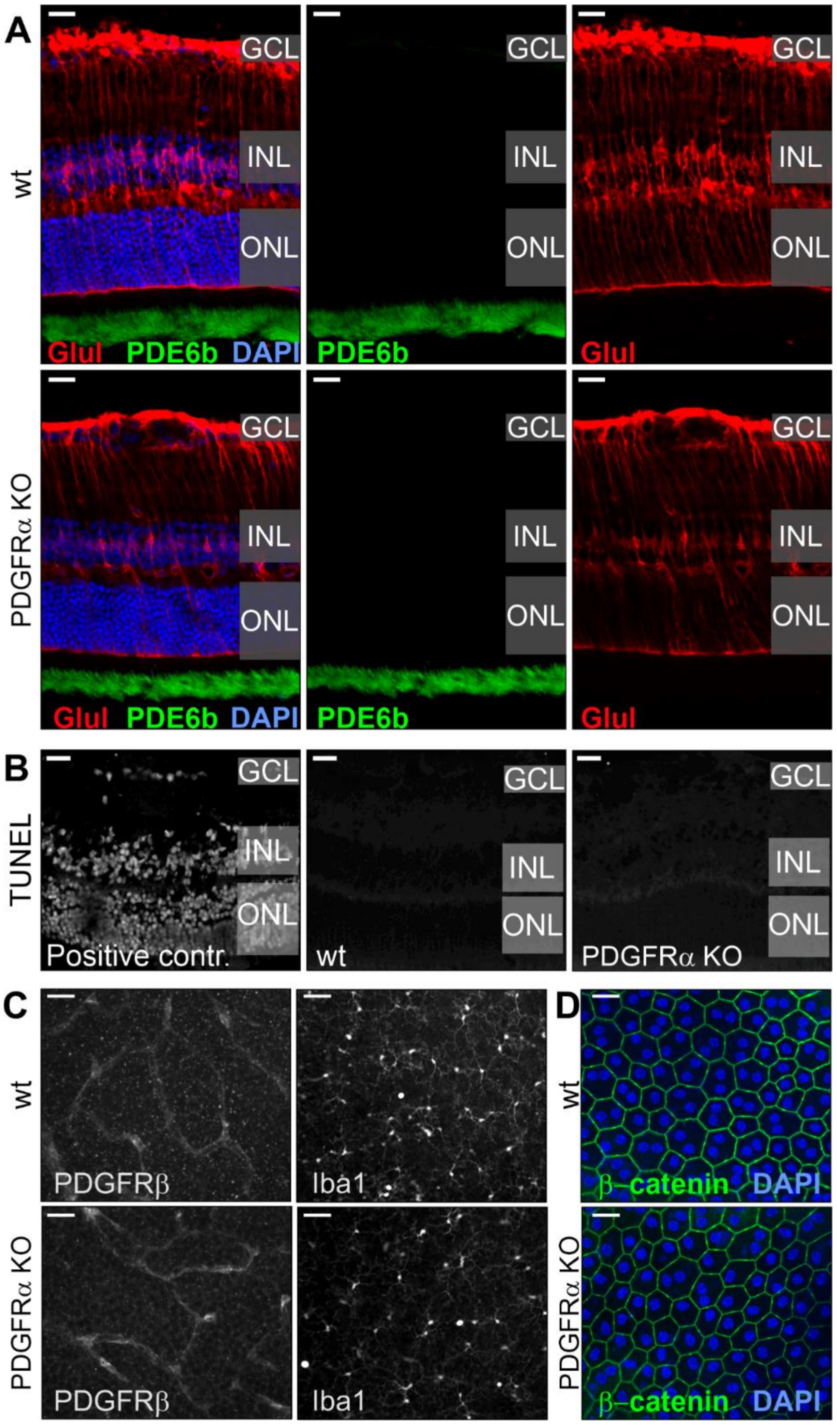

Figure S2. Immunoreactivity of additional cell markers in Müller cell-specific PDGFRα retinae.
(A) Slices from wildtype (wt) and PDGFRα KO eyes were immunostained for the Müller glia marker glutamine synthetase (Glul) and PDE6b, a marker for rod outer segments. The intensity of labeling of Müller glia and rods showed a similar pattern between the both genotypes. **(B)** TUNEL staining was performed to identify cells that may undergo cell death via the apoptosis pathway upon ablation of PDGFRα. While almost every nucleus in the three nuclear layers of the retina were positive as a result of DNAse pretreatment (positive contr.), no TUNEL-positive nuclei were found in wildtype or PDGFRα KO retinae. **(C)** Retinae were flatmounted and labeled using a pericyte (PDGFRβ) and a microglia-macrophage marker (Iba1). No major changes in pericytes and microglia were observed in the PDGFRα KO mice in comparison to wildtypes controls. **(D)** Flatmounted RPE-choroid from wt and PDGFRα KO eyes were labeled for β-catenin, to analyze retinal pigment epithelium (RPE) integrity. No major changes were observed between the two genotypes. Scale bars, 20 µm.
